# Application of Mobile Health Technologies Aimed at Salt Reduction: Systematic Review

**DOI:** 10.2196/13250

**Published:** 2019-04-17

**Authors:** Shahmir H Ali, Rong Luo, Yuan Li, Xiangjun Liu, Chengyao Tang, Puhong Zhang

**Affiliations:** 1 Krieger School of Arts and Sciences Johns Hopkins University Baltimore, MD United States; 2 The George Institute for Global Health Peking University Health Science Center Beijing China; 3 Faculty of Medicine University of New South Wales Sydney Australia; 4 School of Health Humanities Peking University Beijing China; 5 Public Health Graduate School of Medicine Osaka University Osaka Japan

**Keywords:** mobile health, sodium, diet, cardiovascular diseases, systematic review

## Abstract

**Background:**

High salt consumption has contributed to the rise of noncommunicable diseases around the world. The application of mobile health (mHealth) technologies has witnessed rapid growth in recent years. However, evidence to support mHealth interventions to confront the challenge of salt reduction has not yet been critically reviewed.

**Objective:**

The aim of this study was to identify, characterize, and evaluate mHealth interventions aimed at salt reduction across the world.

**Methods:**

A systematic search of studies in English or Chinese language published from January 1, 1992 to July 31, 2017 was conducted using 4 English databases (PubMed, MEDLINE, Global Health, and Cochrane) and 3 Chinese databases (Wanfang, China Science and Technology Journal, and China National Knowledge of Infrastructure). All studies directly using mobile technologies in health care with a primary or secondary objective of reducing dietary salt consumption were included.

**Results:**

A total of 1609 articles were found using the search strategy, with 11 full articles (8 English and 3 Chinese) being included for data extraction, including 11 interventional studies. Overall, few high-quality interventions were identified. Most interventions were limited by small study population sample sizes, lack of control groups, and short follow-up times, all of which were obstacles in generating long-term scalable approaches. Most interventions employed short message service as a platform for mHealth interventions, whereas some innovative mHealth technologies were also explored. Most interventions had a primary focus of improving awareness of dietary salt consumption. The outcome variables used to measure intervention effectiveness included 24-hour urinary sodium excretion, spot urine sampling, dietary records, and indirect behavior or knowledge indicators targeting salt consumption. Although most interventions displayed positive outcome results, none of them provided reliable evidence to evaluate the effectiveness of salt reduction.

**Conclusions:**

Salt reduction in mHealth initiatives remains relatively unexplored; however, studies that did intervene on salt-reduction show the potential of mHealth as an effective intervention method. We provide 3 recommendations for future mHealth interventions in salt reduction—(1) increased use of new, innovative, and interactive mHealth technologies; (2) development of mHealth interventions with primary prevention measures and goals of salt reduction; and (3) large-scale, rigorously designed, and object-targeted clinical trials of mHealth interventions with appropriate quantitative outcome variables, in particular 24-hour urine sodium.

## Introduction

### Salt Consumption and Its Impact on Health

Noncommunicable diseases (NCDs) have become one of the leading causes of disease globally, and their impact on the global health burden has recently surpassed that of infectious diseases [1,2]. In fact, the burden of NCDs in developing countries seems to be worsening, with projections suggesting that by 2020, 7 out of 10 deaths in developing countries will be a result of NCDs [3]. Among the various dietary determinants of detrimental health outcomes, high salt intake has been noted as a key contributing factor for the prevalence of NCDs around the world. High-salt diets are linked to elevated blood pressure, a major risk factor for heart diseases and stroke, which in turn are among the leading causes of death worldwide [4-7]. In fact, salt reduction has been identified as 1 of the 5 priority interventions in response to the global NCD crisis [8]. Developing countries, in particular, have experienced a notable rise of excessive salt intake [9], with Uzbekistan, Turkmenistan, Thailand, China, and Vietnam among the countries falling within the top 20 highest consumers of salt in 2010 [10].

### Impact of Salt-Reduction Initiatives on Disease Burden of Noncommunicable Diseases

The millennium development goals have emphasized an urgent need for adequate and equitable health services through the improvement of global health systems [11]. NCDs such as cardiovascular diseases (CVDs) have placed an increasing burden on the developing world, with 80% of all CVD-related deaths occurring in low- to middle-income countries [12]. Insufficiencies in health services are also reflected in the notable significance of health equity challenges in low-income countries [13]. Reducing dietary salt intakes is increasingly being foregrounded as a highly effective and implementable measure of reducing the burden of NCDs globally. Research has shown that a 15% reduction in population salt intake could prevent 8.5 million cardiovascular deaths over 10 years in 23 developing countries and result in major cost savings [14]. Following the 2011 Moscow global ministerial conference, the United Nations identified salt reduction as among its 3 key cost-effective priority actions to reduce the risk of NCDs [15]. In 2013, the World Health Organization (WHO) identified a 30% mean reduction of population salt intake as one of its 9 global targets of reducing NCDs [16].

### Salt Reduction Interventions Globally

Globally, more countries are now conducting various interventions and formulating strategies to reduce population-wide salt intake to prevent and control NCDs. The main implementation strategies for salt reduction are food reformulation, consumer education, front-of-pack labeling, interventions in public institution settings (such as schools, hospitals, and the workplace), and taxation [17]. Almost all countries are multifaceted in their approach to dietary salt reduction, and consumer awareness and education activities are often used in conjunction with other salt-reduction interventions. However, different countries may need different salt-reduction strategies and interventions [17]. For example, in most developed countries, salt reduction can be achieved by reduction in the amount of salt added to food by the food industry. In contrast, in some developing countries such as China, where salt added by consumers during cooking or in sauces plays a bigger role in population salt intake, salt-reduction interventions tend to target settings such as schools, workplaces, or hospitals and focus more on public health campaigns to encourage consumers to use less salt [17]. Although many of the traditional approaches to salt reduction have been evaluated and significant reductions in salt intake observed [18], most of these interventions are resource intensive and limited in population reach. Therefore, with the rapid change of technology, mobile health (mHealth) represents a potential platform that can be used to address some of these challenges.

### Rapid Growth of Mobile Technologies and Development of Mobile Health

The mobile technologies sector has been a field of great innovation and improvement in recent years. The number of mobile phone users are estimated to have surpassed 5 billion in 2017 [19], and in China alone, there are an estimated 788 million people who access internet via mobile phones (or about 98.3% of the total Chinese internet-using population) as of June 2018 [20]. Given this potential, mobile technologies have played an increasingly significant role in the health sector. mHealth has been defined as “medical and public health practice supported by mobile devices, such as mobile phones, patient monitoring devices, personal digital assistants (PDAs), and other wireless devices” [21]. There are several key functions of mHealth, including health education, data collection, electronic data systems, and clinical decision support systems [22-24].

### Aims and Objectives

Despite the strong advocacy of mHealth as an innovative tool for improving both quality and access of health care [25-27], there remains a great need of continued systematic academic evaluations of its direct impact on key health outcomes [28-33]. Can mHealth be used as an innovative and effective long-term means of addressing global salt-reduction objectives? Currently there is no systematic review yet to describe the use of mHealth apps for salt-reduction purposes. The specific aims of this systematic review are to (1) characterize mHealth developments aimed at dietary salt reduction across the world, (2) evaluate the impact and effectiveness of mHealth interventions focused on salt reduction, and (3) identify the success factors and gaps in mHealth intervention development and evaluation on salt reduction that need to be addressed in the future.

## Methods

### Database Search

We followed the methods detailed in a peer-reviewed systematic review protocol that is registered with International Prospective Register of Systematic Reviews (PROSPERO: CRD42017075361). A systematic search of the literature in both Chinese and English published from January 1, 1992 to July 31, 2017 was performed following the preferred reporting items for systematic reviews and meta-analyses guidelines [34,35] using 4 English electronic databases—PubMed, MEDLINE, Global Health, and Cochrane; and 3 Chinese databases—Wanfang Database, Chinese Science and Technology Journal Database, and China National Knowledge of Infrastructure. We also searched for registered trials in the WHO International Clinical Trials Registry Platform and Clinicaltrial.gov, and some gray literature sources (notably a manual search of reports and government documents). The search equation is built around free-text words referring to mHealth and salt reduction. Keywords used in the English database search strategy include the following: mobile phone, cell phone, mHealth, text message, personal digital assistants, salt consumption, salt reduction, salt management, sodium intake, sodium restriction, and sodium education. Keywords used in the Chinese database search strategy include the following: “Shou Ji” (mobile phone, cell phone), “Yi Dong Jiang Kang” (mHealth), “Yi Dong Yi Liao” (mobile medical), “Yan” (salt), and “Na” (sodium). Multimedia Appendix 1 lists the detailed search strategy for each database.

### Inclusion and Exclusion Criteria

Articles from all countries and with study populations of all ages, genders, and ethnicities were within the scope of this review. A study (with full-text available) was included under the condition that (1) it must include some form of measurement or incorporation of salt education, testing, or consumption data; (2) the study has an mHealth component, with mHealth being defined as “medical and public health practice supported by mobile devices, such as mobile phones, patient monitoring devices, personal digital assistants (PDAs), and other wireless devices” [21]; (3) the types of studies are randomized controlled trials (RCTs), noncontrolled trials, observational studies, qualitative interventional and quasi-experimental (QE) studies, and review studies; (4) it should be written in English or Chinese; and (5) it should be published from 1992 to 2017. A study was excluded if (1) no full-text was available, (2) the full-text was neither in English nor Chinese, (3) it did not include any significant salt-reduction-oriented component, (4) it used exclusively internet of computer or Web-based interventions, (5) it was not conducted on humans, and (6) it focused on biochemistry or microbiology.

### Study Selection

Using the criteria, 2 independent reviewers conducted the abstract screening process for the English database results, and 2 independent reviewers conducted the abstract screening process for the Chinese database results. Full-text reviews were then conducted based on the results of the abstract screening process using the exclusion criteria; 2 independent reviewers reviewed the results from the English database abstract reviews and 2 independent reviewers reviewed the results from the Chinese database abstract reviews. The determinations made by the independent reviewers were then reconciled into a collective decision; if the 2 reviewers were unable to come to a consensus on the relevance of a particular study, then a third independent reviewer was asked to provide his or her input.

### Data Extraction

A spreadsheet was developed to extract key data points from the filtered studies. The extraction sheet was pilot-tested on 10 randomly selected records and refined. Information extracted from each study consisted of characteristics of study participants (including age, sex, disease, and setting of the study), study design, inclusion and exclusion criteria (including type of mHealth device used), and relevant outcome measurements (salt-consumption behavior, salt intake, urinary sodium, and usability of the select mHealth device). A meta-analytic statistical comparison was unable to be performed because of the small study sample size and diversity of outcome indicators. All reviewers reached a consensus on all key definitions and interpretations of the data extraction form before the extraction process was commenced. Overall, 2 reviewers independently extracted data from the filtered English studies, and 2 reviewers independently extracted data from the filtered Chinese studies. All extractions were cross-reviewed, and any disagreements were deliberated upon following which, a consensus was reached. If they were unable to reach a consensus, a third reviewer was brought in to provide a definitive input.

### Quality Assessment

Given the overall low methodological quality of QE studies and lack of mature quality assessment tools, we only conducted quality assessment for RCTs. For RCTs, methodological quality was assessed using the Cochrane Risk of Bias Assessment Tool. Specifically, the criteria used to evaluate each study included the random sequence generation, allocation concealment, blinding of participants, personnel and outcome assessors, incomplete outcome data, selective outcome reporting, and other sources of bias. In addition, 2 reviewers independently conducted a quality assessment for each study, and any discrepancies in judgment were discussed and resolved. If discrepancies remained, a third reviewer was invited to make a final judgment.

## Results

### Included Studies

**Figure 1:**
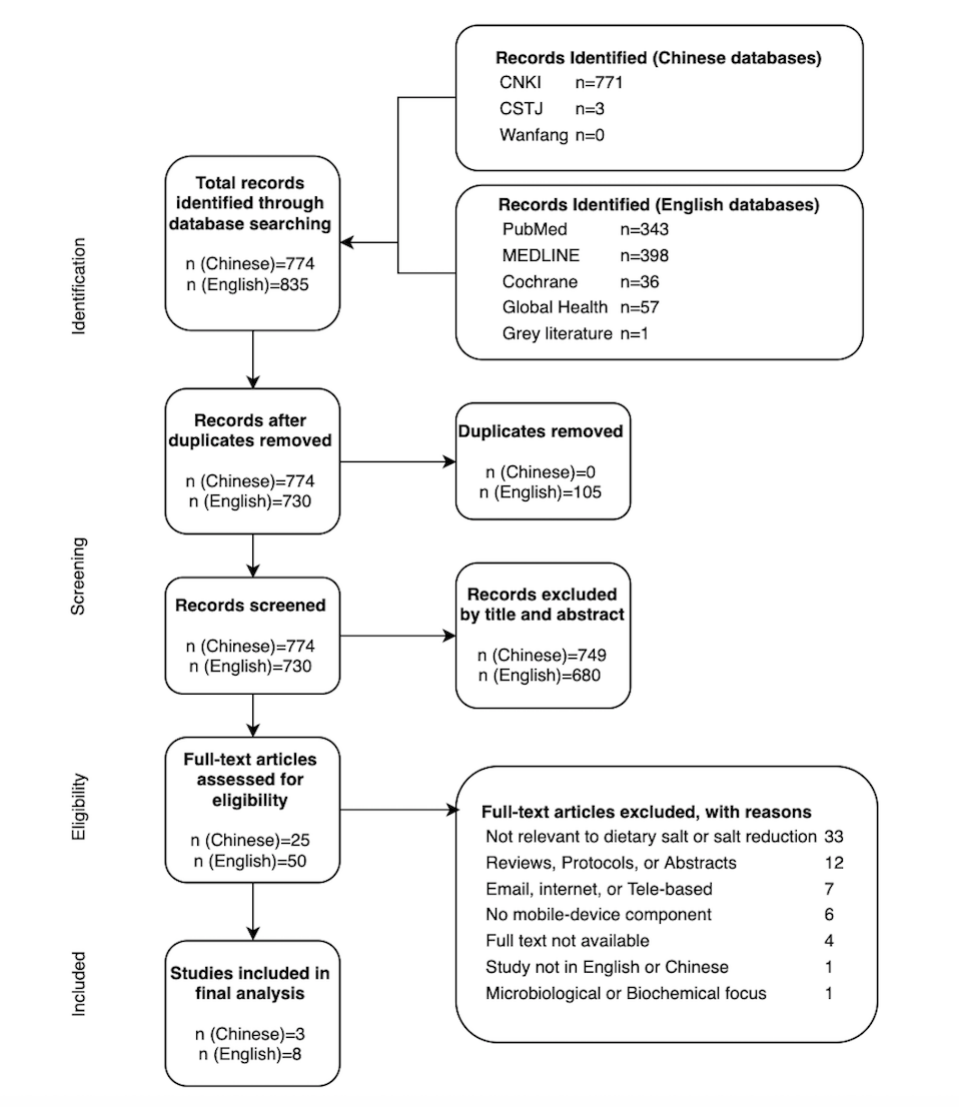
Study flowchart. CNKI: China National Knowledge Infrastructure; CSTJ: China Science and Technology Journal.

We retrieved 1609 articles from the Chinese and English search terms (835 through English search terms and 774 through Chinese search terms), 75 of which were collectively selected for full-text review (50 from the English search and 25 from the Chinese search). The 64 articles were excluded for the reasons specified in Figure 1.

### Study Characteristics

The 11 included studies and their key characteristics are summarized in Table 1. Of these, 6 were RCTs [36-41] and 5 were QE studies [42-46]. Overall, 64% (n=7/11) studies had small sample sizes (had <100 participants), and 73% (8/11) had short follow-up time (<3 months in duration). Although all interventional studies directly evaluated changes in a salt-intake-related variable following an mHealth intervention, only 3 studies had an explicit primary objective of reducing dietary salt intake [36,38,46].

**Table 1:** Summary of key characteristics of included studies (n=11).

Study (year)	Country	Study design	Study population	Intervention duration	Primary intervention target
Eyles et al (2017) [36]	New Zealand	RCT^a^	66 cardiovascular disease patients	4 weeks	Use the app to make lower salt choices
Zhang et al (2017) [37]	China	RCT	72 patients with primary hypertension	3 months	Use the app to improve follow-up efficiency
Ipjian (2016) [38]	United States	RCT	33 adults with mobile phones	4 weeks	Use the app to change dietary, including less sodium intake
Golshahi et al (2015) [39]	Iran	RCT	180 hypertensive patients	6 months	Use SMS^b^ for hypertension self-care, including vegetable intake and blood pressure
Yu et al (2013) [40]	China	RCT	385 community residents	12 months	Use SMS to improve peoples’ knowledge, attitude, practice levels on noncommunicable diseases prevention and treatment
Chen et al (2008) [41]	China	RCT	762 patients with metabolic syndrome	2 years	Use SMS to inform medicine and nutrition information
Lee et al (2017) [42]	South Korea	QE^c^	33 high school students	3 months	Use the app to monitor dietary intake, including various nutrients
Radhakrishnan et al (2016) [43]	United States	QE	27 heart failure patients with minimal cognitive or physical impairment	4 weeks	Use game to improve disease knowledge, self-management, and behavior
Ahn et al (2016) [44]	South Korea	QE	26 patients with diabetes	1 month	Use program or app for nutritional management and dietary change
Huang (2013) [45]	China	QE	165 hypertension patients	3 months	Use SMS and app to improve awareness and control of hypertension
Nundy et al (2013) [46]	United States	QE	15 African American adults with Acute decompensated heart failure	30 days	Use SMS to provide self-care reminders and patient education on diet, symptom recognition, and health care navigation

^a^RCT: randomized controlled trial.

^b^SMS: short message service.

^c^QE: quasi-experimental.

A summary of the mHealth technologies used across the studies is displayed in Table 2. Short message service (SMS) was the most common form of mHealth intervention (n=6) [36,39,40,41,45,46]. Some studies incorporated a multimethod mHealth intervention approach, including combining SMS with QQ (Tencent) - an instant communication software in China similar to Facebook and Twitter [45] - and combining SMS with educational pamphlets [41]. Interactive mobile phone apps were used by 5 studies [36-38,42,44]. Of the intervention studies, most (n=8) had a primary focus of improving awareness and education on dietary salt consumption. Direct nutritional guidance by medical experts or passive dissemination of specific strategies or background information pertaining to salt-reduction was commonly employed [37,39-41,45,46]. Moreover, 1 study explored a more active form of health promotion by using a mobile phone app to allow consumers to scan packaged foods and beverages for nutritional information as well as low-salt alternatives [36]. Overall, 4 studies focused on disease management or the monitoring of diet trends [37,38,42,44], all of which involved a component of written input of daily consumption information. Moreover, 2 of the studies also involved photographic input of daily consumption information [37,44], 2 studies involved an audio component [37,42], and 1 study involved a video component [37]. For the function of mHealth interventions, 7 studies used mHealth for health education and awareness to patients [36,37,39-41,43,46], 3 studies used apps to monitor behaviors [38,42,44], and 1 study used SMS to provide self-care reminders to patients [45].

The majority of the studies were conducted in an urban area (n=9) [36-40,42,43,45,46], whereas the other 2 studies did not mention about rural or urban areas. Overall, 5 studies occurred in community settings [36,38,40,42,44], whereas 6 occurred in hospital settings [37,39,41,43,45,46]. The most common disease focus was hypertension (n=3) [37,39,45], whereas other studies centered around heart failure (n=2) [43,46], CVDs (n=1) [36], diabetes (n=1) [44], metabolic syndrome (n=1) [41], and any NCDs (n=1) [40].

### Effectiveness of Mobile Health

The salt-reduction-related outcome variables employed by the studies with RCT or QE study designs are summarized in Table 3.

**Table 2:** Technologies used in the randomized controlled trials and quasi-experimental studies (n=11). A checkmark indicates that the technology was observed in the study, while an em dash indicates that it was not.

Study	Short message service	App-based	Others (eg, game device)	Web-based	In-person
Eyles et al [36]	✓	✓	—	—	—
Golshahi et al [39]	✓	—	—	—	—
Yu et al [40]	✓	—	—	—	—
Chen et al [41]	✓	—	—	—	✓
Huang [45]	✓	—	—	✓	—
Nundy et al [46]	✓	—	—	—	—
Zhang et al [37]	—	✓	—	—	—
Ipjian and Johnston [38]	—	✓	—	—	—
Lee et al [42]	—	✓	—	—	—
Ahn et al [43]	—	✓	—	✓	—
Radhakrishnan et al [43]	—	—	✓	—	—
Total	6	5	1	2	1

**Table 3:** Primary outcome and outcome variables employed by studies categorized by study design and if with successful salt reduction (n=11).

Study design		Outcome variables^a^			
		24-hour urine (mg/24 hours)	Spot urine (mg/L)	Dietary record	Behavior or knowledge indicators
**Randomized controlled trial: Is salt-reduction or intake a primary outcome?**					
	Yes (n=2)	0	2 [36,38]	0	1 [36]
	No (n=4)	0	0	2 [39,41]	2 [37,40]
**Quasi-experimental study: Is salt-reduction or intake a primary outcome?**					
	Yes (n=0)	0	0	0	0
	No (n=5)	0	0	3 [42,44,45]	3 [43,44,46]
Total		0	2	5	6

^a^A study might employ multiple outcome variables to evaluate the effectiveness on salt reduction, so that the total number of outcome variables might be larger than the number of studies in the same row.

**Table 4:** Summary of effectiveness results (among the 11 randomized controlled trials and quasi-experimental studies).

Outcome variable category	Studies with positive result^a^, n (%)
Spot urine	2 (100) [36,38]
Dietary record	4 (80) [41,42,44,45]
Behavior or knowledge indicators	2 (33) [40,46]
Total	8 (73)

^a^A study outcome was regarded as positive if the postintervention study population recorded statistically significant decreases in salt intake or improvements in behavior or knowledge indicators implying the reduction of salt consumption.

The primary categories examined 24-hour urinary sodium excretion, spot urine sampling, dietary records, and indirect behavior or knowledge indicators targeting salt consumption. The dietary record category included any self-reported measurements of daily salt intake. The behavior or knowledge indicators category reflected study-specific measurements of changes in either behavior or knowledge variables that linked with sodium consumption tendencies. The results of the outcome variables are summarized in Table 4.

Overall, a majority of the studies (8/11, 73%) showed a positive result in the salt consumption-related outcome indicators examined.

No RCT or QE study evaluated the effectiveness of salt reduction using the 24-hour urine collection method. Dietary sodium consumption records were measured through 24-hour dietary recalls [42,44] as well as changes in participants reporting daily salt consumption above 10 g per day [41] and below 6 g per day [40,45]. Most studies displayed positive results; self-reported reductions in the frequency of days of high salt consumption (more than 10 g/day) [41] as observed, along with increased self-reporting of salt consumption of less than 6 g per day [40,45]. Moreover, 1 intervention noted a poststudy daily salt intake decrease of approximately 0.8 g per day [42]. Salt consumption-related behavior or knowledge outcome indicators included Self-Care Heart Failure Index (SCHFI) [46]; survey questions on whether participants ate salty foods [44,39]; salt content of household packaged food purchases [36]; self-identified salt consumption patterns of either “light,” “medium,” or “heavy” [37]; and self-motivation to reduce salt consumption. Overall, 4 studies found no statistically significant difference in salt consumption behavior post intervention [36,37,39,44]. Among studies with positive results, 1 study showed improvements in the SCHFI index questions on maintenance of low-salt diets and reductions in salt intake [46], whereas another study found that 84% of participants agreed the mHealth game used in the intervention motivated them to restrict their salt intake [43]. Indeed, overall, given the large discrepancies in study methodology and mHealth technologies employed within this study, systematically and conclusively identifying key success factors in mHealth interventions targeting salt consumption remain difficult.

### Quality Assessment

Using the Cochrane Risk of Bias Tool, the majority of the bias risk criteria for the included RCTs remained either unclear or low (Multimedia Appendix 2). The quality of 5 QE studies was not assessed.

## Discussion

### Principal Findings

mHealth is becoming increasingly popular around the world as the information technology, telecommunication, and health sectors continue to experience waves of innovation. With this progress, the development of mHealth interventions and its evaluation have also become increasingly prominent. In this comprehensive systematic review, we examined the application of mHealth technologies aimed at salt reduction in the published studies and registered trials. We particularly focused on interventional studies but also examined studies of other methodologies examining mHealth in this area. Most (7/11, 64%) studies indicated that mHealth interventions indeed had a positive effect on salt reduction; however, these studies also had very small sample sizes and short intervention periods, whereas none of the RCTs or QEs used the 24-hour urinary excretion measurement method (the gold standard of evaluating salt intake). The overall number of published papers (n=11) remained extremely small, 10 of which (91%) were published on or after 2013, indicating that the evaluation of mHealth technologies for the purposes of dietary salt reduction is indeed at an early stage. A vast majority of the studies examined salt reduction as a mere component of a broader investigation of other macronutrients—only 2 studies focused exclusively on salt reduction [36,38], and few studies had clear objectives and goals of salt reduction. Thus, there is need for greater and more specific focused mHealth interventional research with clear objectives and goals of salt reduction.

SMS platforms were identified as the most common form of mHealth intervention in salt reduction. However, this review also identified a number of innovative user-engagement methods of mHealth technology that hold potential for further exploration and research. For example, 1 study involved the design of a mobile-device video game that incorporated salt reduction promotion [43], whereas another study examined a mobile app that allowed users to actively scan food products and receive nutritional information as well as targeted low-salt alternatives [36]. Some studies incorporated mobile phone programs with specific regional relevance; the Chinese social media apps QQ and WeChat were used in 2 studies conducted in China [37,45]. Popular global social media platforms such as Facebook, Twitter, and YouTube, thus, also hold the potential to be used as tools of health, given the abilities of such platforms to disseminate information quickly, reach broader audiences, customize health messages for specific groups, and encourage interaction and engagement [47,48]. The intensity of user engagement across the mHealth technologies and interventions examined remained relatively minimal and was commonly limited to instructive and reminder-oriented forms of health promotion. Indeed, there is a greater need for further research on various mHealth interventions to better gauge the true potential of mHealth technologies to achieve salt-reduction goals.

In this review, most studies (8/11, 73%) focused on populations diagnosed with specific salt-related diseases. Many of the salt-reduction initiatives examined tended to be part of broad treatment plans for those already experiencing diseases such as hypertension and cardiovascular illnesses. Nonetheless, some studies examined much broader subgroups, including healthy adults [38], high school students [42], and community residents [40]. Thus, given that high salt intake is an issue identified across multiple general subpopulations [4,5], this review identifies a strong need for mHealth-based salt reduction interventions aimed at disease prevention among healthy populations as opposed to simply disease management as well as targeted interventions to better engage with a wider spectrum of subpopulations.

With respect to overall effectiveness of mHealth as an interventional tool for dietary salt reduction, given the significant heterogeneity in intervention designs and outcome indicators across the interventional studies, no decisive conclusions pertaining to effectiveness have currently been reached. A majority of the outcome measures focused on qualitative or survey-based indicators. Ultimately, the emphasis on qualitative indicators in the included studies indicates a very strong need for studies that use more quantitative and standardizable measurement indicators. The 24-hour urinary sodium excretion method is considered the gold standard in the measurement of sodium intake [49]. Single 24-hour urinary sodium readings are often subject to high levels of discrepancies between individuals in a population; thus, consecutive 24-hour urinary sodium collection readings over several days can reduce the impact of intraindividual differences. However, 24-hour urinary sodium collection carries a high participant burden of compliance; thus, many studies have to rely on alternative sodium intake measurement options—such as spot urine sampling. None of the interventional studies in the final study sample employed 24-hour urinary sodium measurements as an outcome indicator, whereas only 2 studies utilized spot urine sampling [36,38], signaling a clear need for further mHealth research evaluating changes in salt consumption using high-quality sodium intake measurements.

It is estimated that a total of 75 countries now have a national salt reduction strategy, with the majority of programs including interventions in public institutions [17,50]. However, across the included studies, China and the United States, the 2 engines of global internet development, are clearly leading the way in mHealth-related interventional research on salt-reduction promotion. We believe that more countries, especially those countries with high dietary salt consumption, will utilize mHealth as an intervention measure to reduce salt consumption for hypertension and CVD prevention and control in the coming years. Likewise, mHealth interventions that can target consumers of salt may particularly benefit countries (such as China) in which the use of large quantities of salt in cooking plays a larger role in population salt intake than food processing and preservation by food industries (which is a more significant problem in countries such as the United States and Japan) [51].

### Quality of the Evidence

Our review found that most interventional studies were limited by small sample sizes, a lack of control groups and reliable salt-intake measurements, and short follow-up times; the external and internal validities of many studies remain low. Thus, the low-quality nature of this study cannot provide substantive evidence for more scaled-up implementations. Therefore, well-designed health interventions and rigorous evaluation of salt-reduction programs will be vital for future scale-up and policy making.

### Limitations

There were several limitations in our review. First, we were unable to conduct a meta-analysis to evaluate effectiveness of mHealth interventions and conduct further analysis of success factors in mHealth intervention development and evaluation on salt reduction because of the significant heterogeneity of outcome indicators and health issues reported from the very limited number of RCTs. Second, only English and Chinese language articles were included, and only 4 English and 3 Chinese databases were examined. Third, this review focused on academic studies in the literature; mHealth is growing in prominence within the commercial world, and not all of this potential health impact has been studied exclusively in academic contexts.

### Conclusions

In summary, despite the growth of mHealth and its potential to achieve salt-reduction goals, evidence for a positive effect on health is still limited. Salt-reduction as a primary focus in mHealth initiatives remains relatively unexplored, and the emphasis on salt reduction is not very strong. However, among the included studies, the use of mHealth interventions has generally led to improvements in salt-reduction-related outcomes (notably changes in sodium from spot-urine sampling and dietary salt consumption records). In general, the methodological quality of the studies included in this systematic review is low. However, there are numerous opportunities for research and implementation of mHealth in salt reduction. Moving forward, we have identified 3 priority areas to improve the mHealth research agenda—(1) increased use of new, innovative, and interactive mHealth technologies (such as mobile phone apps) in the context of salt reduction; (2) development of mHealth interventions with primary prevention measures and goals of salt reduction; and (3) large-scale, rigorously designed, and object-targeted clinical trials of mHealth interventions aimed at salt reduction with appropriate quantitative outcome variables, in particular, 24-hour urinary sodium.

## Appendix

Multimedia Appendix 1 Search strategy.

Multimedia Appendix 2 Quality assessment of included randomized controlled trials (using Cochrane Risk of Bias Tool).

## Abbreviations

CVDs: cardiovascular diseases
mHealth: mobile health
NCDs: noncommunicable diseases
QE: quasi-experimental
RCT: randomized controlled trial
SCHFI: Self-Care Heart Failure Index
SMS: short message service
WHO: World Health Organization
