# Supplementary figures and images for: Application of Mobile Health Technologies Aimed at Salt Reduction: Systematic Review
Source: JMIR Mhealth Uhealth. 2019 Apr 17;7(4):e13250. doi: 10.2196/13250 (PMC6492062; doi:10.2196/13250)

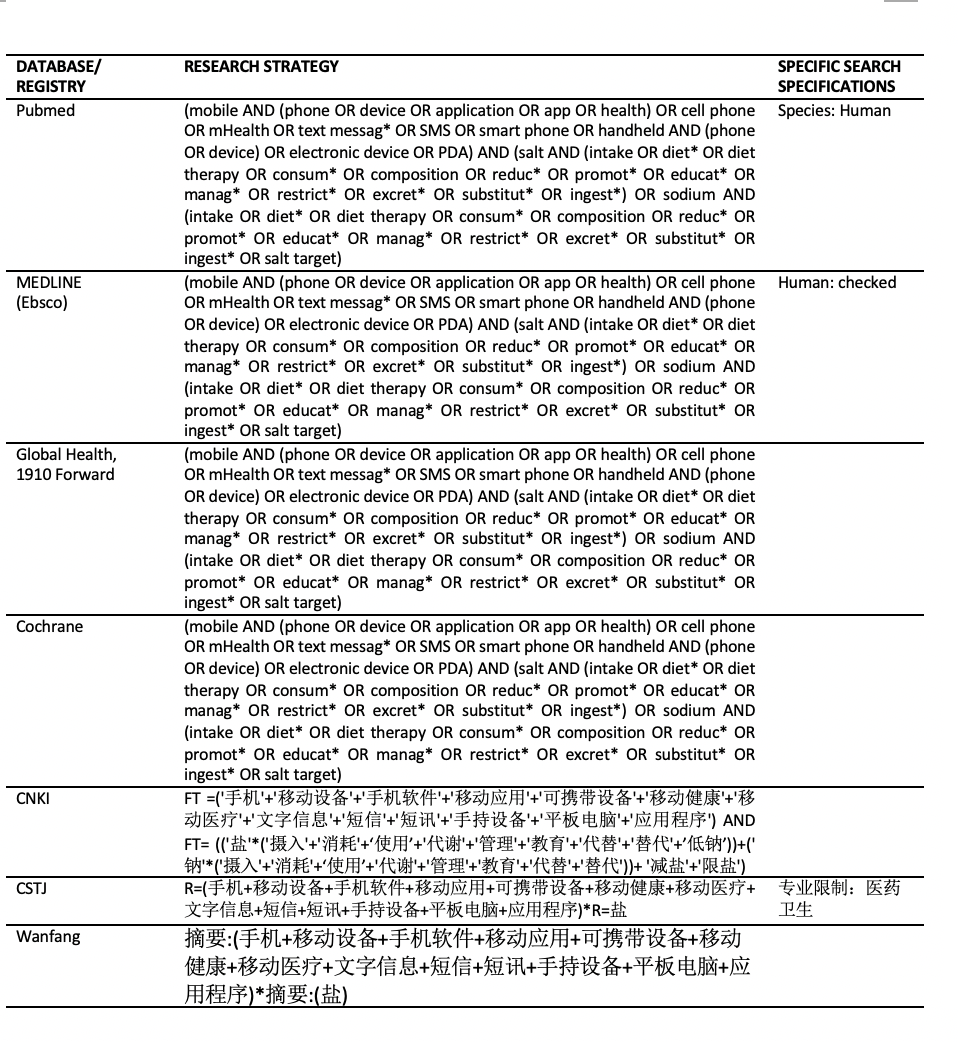

Supplement: Multimedia Appendix 1 [file mhealth_v7i4e13250_app1.png]

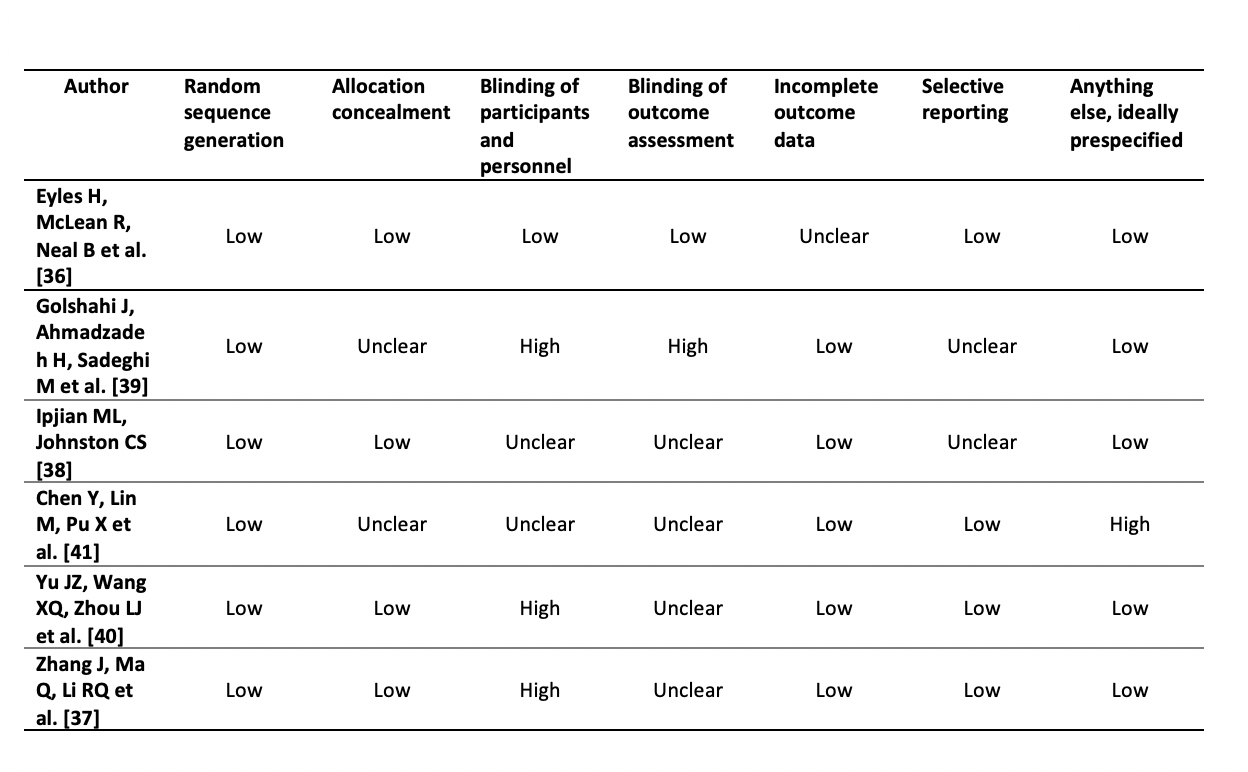

Supplement: Multimedia Appendix 2 [file mhealth_v7i4e13250_app2.png]
